# Anthropological study on Chagas Disease: Sociocultural construction of illness and embodiment of health barriers in Bolivian migrants in Rome, Italy

**DOI:** 10.1371/journal.pone.0240831

**Published:** 2020-10-16

**Authors:** Miriam Castaldo, Andrea Cavani, Maria Concetta Segneri, Gianfranco Costanzo, Concetta Mirisola, Rosalia Marrone

**Affiliations:** 1 Department of Mental Health - Medical Anthropological Unit, National Institute for Health, Migration and Poverty (INMP), Rome, Italy; 2 Scientific Coordination Unit, National Institute for Health, Migration and Poverty (NIHMP), Rome, Italy; 3 Medical Directorate, National Institute for Health, Migration and Poverty (INMP), Rome, Italy; 4 INMP Directorate, National Institute for Health, Migration and Poverty (INMP), Rome, Italy; 5 Multispecialty and Medical Professions Department, National Institute for Health, Migration and Poverty (INMP), Rome, Italy; Graduate School of Public Health and Health Policy, City University of New York, UNITED STATES

## Abstract

**Introduction:**

Chagas Disease (CD) is endemic in many Latin-American countries, Bolivia in particular. It is now spreading in Italy as a host country for transcontinental migrants and becoming an emerging health problem. This anthropological action–research, as part of a wider medical project on Neglected Tropical Diseases, has the purpose of analyzing the sociocultural construction of CD and its representation in Bolivian people living in Rome as well as barriers, such as the stigma about the illness, to access the National Health Service for those potentially affected.

**Methods:**

The ethnographic study was carried out from 2016 to 2018 by a medical anthropologist at the National Institute for Health, Migration and Poverty (INMP) on 72 Bolivian migrants (47 women and 25 men) living in Rome. The study was carried out through: a territorial mapping of Bolivian networks and communities aimed at recruiting people, participant observation, and application of semi-structured and unstructured interviews. The interviews were hold in Spanish and proposed to all participants before or during medical examination, or during events organized by the Bolivian community in Rome. The interview consisted of 16 items and covered four macro areas: personal and migration history, health status, access to the Italian National Health Service and knowledge about CD; plus 5 items for those who received a diagnosis of Chagas Disease in Italy.

**Results:**

The sociocultural construction and the deep stigma about the illness built by participants and their families could hinder both diagnosis and treatment. Institutional barriers also contributed to reduce adherence to screening tests: often, opening hours of the outpatient clinic were incompatible with participants’ precarious employments. To guarantee participant’s access to public health services and their adherence to the diagnostic protocol, we implemented a profound revision of our cultural and institutional approach to them.

**Conclusions:**

The analysis evidenced the limitations of the conventional approach applied by the Italian National Health Service to this migrant community, such as the absence of socio-cultural and linguistics competences that can help understanding patients’ perception and representation of the illness. The multidisciplinary approach instead—with clinicians using the ethnographic results to adjust their work to the participants’ needs—was a successful attempt to ensure therapeutic alliance.

## Introduction

Chagas Disease (CD) is one of the so-called Neglected Tropical Diseases (NTDs), mainly rooted in rural areas of the del American continent and spread through internal and external migration [1, 2]. NTDs affect populations living in poverty, without adequate sanitation and access to clean water or proper ways to dispose of human waste and in close contact with infectious vectors, domestic animals and livestock [1]. CD seems to be a metaphor for those social inequalities and poverty conditions that are produced in the contexts of origin and later reproduced in the migratory hosting countries: therefore, it is possible to state that CD can be eligible to receive a sort of archeological analysis.

CD has now become a global health phenomenon affecting a significant number of people, also in non-endemic countries—particularly southern European countries such as Spain and Italy [3–7]–where it is generally hardly diagnosed and therefore underestimated [6–9]. Chagas spread in Europe and mainly in Italy occurs through different non-vectorial routes. It is due to population movements, blood transfusion or organ transplant, childbirth, adoptees people visiting friends and relatives and, also, by laboratory-accident transmission. It is common for European doctors to have little or no experience with the detection and management of Chagas disease [7]. Mainly because of the difficulty of migrants to access screening and prevention programs, and because CD is not included in traditional diagnostic-therapeutic pathways [7]. This disease, however, can be reactivated in immunosuppressed patients, giving rise to serious and potentially fatal clinical pictures [4–6].

Although epidemiologic data about CD presence in Italy are very limited, approximately 6–12,000 estimated cases [10–12], the highest prevalence is estimated among migrants from Bolivia [4].

The triatomine bug usually called *vinchuga* or *chinche* by the inhabitants of Latin America is the main vehicle for the protozoan parasite *Trypanosoma Cruzi* (T. *Cruzi)* that ultimately causes Chagas Disease. Vinchuga is widely spread in Latin America, especially in rural areas (other typical names are *chipo*, *pito*, *barbeiro*, *chichaguaz*). In both endemic and non-endemic countries, inter-human transmission of *CD* can also occur by means of blood transfusion and tissue transplantation, or congenitally from mother to child [13–15]. Once infected, it is possible to be asymptomatic for years before showing heterogeneous symptoms involving cardiac, gastrointestinal and central nervous system [2, 16].

The World Health Organization (WHO) estimates that about 6,000,000 people are infected by *T*. *Cruzi* throughout the world, 62% of whom live in countries of the Southern Cone, Bolivia in particular, where at least 6% of its population is considered affected [17, 18]. In 1999, Bolivia launched the Chagas National Program (“*Programa Nacional de Chagas*” in Spanish) trying, in this way, to sanitize all the endemic municipalities of the country; finally, in 2006 the eradication was qualified as a national priority with Bolivia’s Chagas Disease Law [19]. To fulfill the purpose, the government offered free diagnosis and treatment in all major cities but without considering the individual and collective sociocultural construction about CD and the etiological and therapeutic “traditional” system based on other knowledge and care systems—the so-called imaginary about Chagas and its vectors in the target population [20–24]. These aspects would have been of great importance for the implementation of health campaigns in terms of prevention and sustainability [25–27] but unfortunately, they were not considered.

Starting from this historical and epidemiological framework, we launched our anthropological research on CD as a part of a project on NTDs: “Strengthening the fight against neglected Tropical Diseases in the migrant population through the use of medical devices”, precisely in consideration of the above-mentioned social and cultural aspects, but also of the political and social contexts from which immigrants come—pervaded by the violence of poverty—that strongly affect the Chagas diagnosis and treatment also in Italy [28, 29]. The challenge of this research, was to keep together the migratory process from the native land to the host country—that also determined the conditions of poverty in Italy—with the Chagas concept that the research target people had of the disease. This by trying not to reduce the disease only to its biophysical dimension [30], but including in the clinical action the perspective of the patient, the one who experiences the disease, who incorporates and represents it [31].

### Research objectives

The main research objectives are: i) to investigate the occurrence of Chagas disease among Bolivian migrants living in Rome; ii) to analyze the socio-cultural disease construction processes and its representation in relation to the migratory condition; iii) to disclose the social impact of the disease; iv) to identify the barriers to access Italian public health services for potentially affected people.

The INMP anthropologist and the infectious disease specialist used both ethnographic and biomedical methods, by sharing and combining them. To achieve the above-mentioned objectives, the steps taken were the following: a) data collection about existing health policies, both in the country of origin as well as in Italy; b) analysis of the correlation between CD and socio-economic conditions, migration status and institutions; c) analysis of the social and political causes of CD production (and reproduction), both in the country of origin as well as in Italy, d) investigation of the collective imaginary about CD.

### Study context

Anthropological research is part of the clinical project "Strengthening the fight against Neglected Tropical Diseases in the migrant population through the use of medical devices", whose general objective to strengthen the fight against NTDs in migrant population from endemic areas living in Rome, through: (i) an estimate of the spread and epidemiological characteristics of some major neglected tropical diseases such as strongyloides and other geohelminthiasis, schistosomiasis, and Chagas disease; (ii) the early identification and early care of affected migrants. The anthropological study, that only focused on CD, was built to contribute to study the processes of spread of neglected diseases, their understanding and explanation, through theoretical-methodological tools. Final aim is to implement local health policies able to combat these diseases, primarily considering them as a social and economic problem, then a political and legal issue and, at last, a health concern. What we report in this work is the transcultural and transdisciplinary approach usually adopted at the INMP when providing health care to specific populations (poor and disadvantaged groups, regularly and irregularly staying foreigners, victims of violence and trade, international protection applicants).

The anthropologist recruited Bolivian patients for the project and interviewed them for the purpose of research; she followed them throughout the whole medical path to establish a relationship based on trust: during the first medical examination when blood tests were prescribed; when these tests were carried out; in the course of other examinations when the diagnosis was communicated. Then if Chagas diagnosis was positive, she contacted the patients and their families by phone during the whole period of hospitalization in which they were subject to instrumental diagnostic tests and CD treatment.

## Methods

### Study design

The ethnographic study was carried out by the medical anthropologist from August 2016 to July 2018. It included participant observation and administration of both semi-structured and unstructured interviews. All communications and interviews were conducted by the first author in Spanish.

In the first phase of the project—that lasted about one year—we mapped the Roman territorial network to detect Bolivian migrants for possible enrolment in the project. In the second phase we contacted the identified people and associations by telephone or e-mail and offered them free enrollment in the clinical project as well as in the anthropological research. In case of a positive answer, we scheduled an appointment at our outpatient clinic.

### Sample

Eligibility criteria for the sampling required: from 18 years old, to be born and have lived in Bolivia before migrating to Italy, not having a previous diagnosis of Chagas. After signing an informed consent, 72 undiagnosed Bolivian migrants living in Rome were recruited by the first author from December 2017 to June 2018 at the outpatient department of National Institute for health, Migrant and Poverty (INMP), in Rome, Italy.

### Procedure: Participant observation and interview

The ethnographic interviews were conducted in fluent Spanish by the first author at the outpatient clinic, whether during the first medical examination or at the communication of the laboratory tests results. The anthropological activity also included participant observation of the relationships between the doctor and the participant or between the doctor and the family members, for example during medical examinations. Furthermore, participant the observation was rigorously applied during each meeting with the participants, and data reported immediately on a notebook. Additional interviews were administered to participants met during events organized by the Bolivian community in Rome (parties, carnival parades and sport games) or at the housing occupations (squats) where some of the participants lived. Interviews (consisting of 21 items) were semi-structured and unstructured, and guided by a list of topic question (e.g. “By what or who can Chagas be caused?”—Investigate magical-religious, socio-cultural, socio-economic aspects -; “What do people you know think about Chagas?”—Investigate aspects of the social and family imaginary; prejudice, possible isolation, contagion -;—If Chagas diseases is diagnosed- “Since you have this disease, how has your life changed?”; “Did you or your family members ever visit the doctor because of Chagas Disease in your country and in Italy?—Analyze costs and social stigma).

Sometimes the topics changed as the formulation of the meaning of illness developed. The interviews lasted about 30–40 minutes, written down directly and no audiotaped. They covered four macro areas: i) personal history; ii) migration history; iii) health status and access to the Italian National Health Service; iv) prevalent knowledge about CD.

Conversation was never forced, to respect feelings and emotions as well as to provide the time needed by each person or family member. Furthermore, before starting the interview, people were asked what language they preferred between Italian or Spanish.

All the interview items are detailed in Table 1.

**Table 1:** Items included in the anthropological interview.

**Life history**	Personal data
	Education
	Marital status
	Employment and socio-economic status in the country of origin
	Current job (contract type, salary, treatment by employers)
	Living condition in Italy and type of accommodation
**Migration history**	Date of arrival in Italy
	Migratory reasons
	Migration experience and legal status in Italy
**Health status and access to the Italian National Health Service**	Perception of the health status
	Access to Italian hospitals
	Access to family doctor
	Health barriers
	Family health history
**Chagas Disease**	Knowledge about the disease
	Personal experience
	Imaginary
	Previous analysis for Chagas disease
	Disease as a perceived stigma in both Bolivia and Italy

We interviewed each participants at least once, a second interview was administered in case of positive diagnosis of CD. In some cases, we had to administer interviews in a fragmented manner (i.e. in the waiting room—when the patient was alone -, or, in presence of the doctor, before, during or after the clinical examination and even during blood tests) because of the regularly short time available for participants to stay at our clinic due to work reasons.

### Data analysis

The interviews were directly transcribed in Spanish, organized and printed for content analysis; field notes written in Spanish on notebooks were analyzed. Content analysis was conducted from September 2018 to January 2019 manually, through the process of *coding* [32, 33], without using any content analysis software.

### Ethical statement

This anthropological study is part of the Tropical Neglected Diseases Project: “Strengthening the fight against neglected Tropical Diseases in the migrant population through the use of medical devices". It was led by the Italian National Institute for Health, Migration and Poverty (INMP) in Rome and approved by the Ethical committee of the Italian Higher Istitute of Health (Istituto Superiore di Sanità—PRE-712/16).

All people involved in the study signed a written consent according to the Declaration of Helsinki [34].

## Results

### Territorial mapping of Bolivian population living in Rome

Out of the number of organizations, associations and institutions contacted, the following are those who responded positively, sending to their Bolivian contacts the proposal to participate in the research. The reached local network was wide and included many people: the Bolivian Embassy, the roman Bolivian community of the “Asociación de la Comunidad Boliviana”, the roman Bolivian community (not belonging to the “Asociación de la Comunidad Boliviana”), other Latin American associations, Italian schools for foreigners, centers for adopted children, Latin American Christian centers in Rome, parishes, nunneries as well as the Latin American Anglican Church, Catholic communities and self—managed housing occupations.

Territorial mapping played an important role in identifying a larger number of Bolivian migrants for enrollment and facilitating their access to our outpatient clinic for CD screening.

### Participants’ data

No drop out was registered, all the people who were asked agreed to be interviewed. We interviewed 72 Bolivian migrants, 47 women (65.3%) and 25 men (34.7%), mainly from the municipalities of Cochabamba, Oruro, Santa Cruz and La Paz, located in central Bolivia. The large presence of women in the research shows the feminization of migration, typical of Latin America, which is also widely analyzed in literature [35–37].

Participants’ age ranged from 16 to over 65 years old (median age 43.3) (Table 2); most people had a medium-high education: 49 people (68.1%) had a high school diploma: 7 of them (9.7%) had a degree, of which 5 were women (6.9%).

**Table 2:** Distribution of selected characteristics for participants in anthropological study on Chagas Disease 2016–2018.

**Age**	**N°**	**%**
16–17	3	4.2
18–34	9	12.5
35–54	50	69.4
55–64	8	11.1
Over 65	2	2.8
**Sex**		
F	47	65.3
M	25	34.7
**Marital Status**		
Married/cohabitee	42	58.3
Unmarried	22	30.6
Widow	2	2.8
Separated/divorced	6	8.3
**Qualification**		
Junior high school diploma	16	22.2
High school diploma	49	68.1
Degree and beyond	7	9.7
**Employments**		
Part time job	17	23.6
Occasional job	3	4.2
Housewife	4	5.6
In search for a new job	9	12.5
Full time job	33	45.8
In search for the first job	1	1.4
No answer	5	6.9
**Works performed**		
Caregiver for elderly/ baby sitter	18	25.0
Domestic helper	23	31.9
Clerk	2	2.8
Unspecified employment	5	6.9
Industrial worker	2	2.8
Construction worker	2	2.8
Teacher/educator	1	1.4
No answer	19	26.4

### Interview administration

The anthropologist had a leading role in recruiting participants both for clinical screening and for the interview. Though the duration of interview administration was between 30 and 40 minutes (mean duration of 35 minutes), few participants agreed to participate in a larger number of encounters aimed at deepening socio-cultural aspects related to the disease. In many cases, the impossibility for participants to stay at our outpatient clinic for the needed time led to a fragmentation of the interviews into multiple encounters.

### Immigration, work and access to healthcare

Bolivians are quite a stable population in Rome: 97.4% of the interviewed had a residence permit and, among them, 48 (66.7%) had lived in Italy for more than 10 years and had a full (33 people– 45.8%) or part time job (17 people– 23.6%) (Table 2).

Migratory projects have multiple roots and result from individual choices as well as from contextual conditions concerning both the attraction for the immigration country and the reasons for leaving the country of origin [38–40]. Below, we analyze women more than men not only because they are more numerous, but also because Bolivian immigration, such as that of other Latin Americans in Italy, is predominantly female. Furthermore, they also are the ones who take care of the whole family remaining in the country of origin, until the possible family reunification in Italy. It is also important to contact and establish a good relationship with them because of the risk of mother-to-child transmission [13–15]. This scenario is undoubtedly a major public health problem, since affected people may ignore CD signs and symptoms and become seriously ill, and even die, without ever receiving a CD diagnosis. Most Bolivian women migrated to Italy alone: in most cases, they followed a female protective network of sisters, cousins and aunts who previously arrived. Conjugal reunification normally occurs after many years. It is interesting to note that 58.3% of the interviewed are married and only 8.3% separated or divorced (Table 2); however, due to the difficulties of separation/divorce, a substantial number of women created new relationships, exclusively with Latin American men, without formally interrupting the previous bonds.

Many of the interviewees lived with their family and friends in rented or subleased houses or in abandoned buildings (Housing Squat) irregularly occupied and managed by urban social movements.

In their country, Bolivian women had employments consistent with their qualification and professional skills; this is why their migratory project is built upon the hope of improving economic income, to help their family and children with a possible employment inherent to their studies. Despite the high educational level, almost half of the Bolivian women in Rome are employed as domestic helper (23 people, 31.9%), babysitters or caregivers for elderly (18 people, 25.0%) (Table 2).

In the domestic and elderly care work field, it is possible to identify intensive processes in the formation of ethnic niches. Common feelings are those of frustration and resignation, due to the awareness that “as migrants” they can only access to a certain range of unskilled and poorly paid employments. Often, they are subjected to violent forms of domestic slavery, preventing them even from being absent for medical examinations, despite their regular contractual condition. Below the illustrative testimonies of two women:

I used to work as a maid, then I had cervical problems and had to quit. Now I work with two kids, but not every day. I always have had a job by the hour. These jobs are the only ones we can do here

(Marta—May 7, 2018).

In Bolivia I used to work with an NGO in the schooling of women and poor people, here I work as caregiver of two elderly people who have Alzheimer’s… and they are also aggressive, I am practically a slave. They never let me go out; I have little time only on Sundays to come for the analysis and the visit. I am here to work, because I have to help my children who are graduating in Bolivia, that is why I do not give up

(Luz—July 8, 2018).

### Social and cultural construction of Chagas Disease: Fear and stigma of poverty

Among the 72 participants interviewed, screening procedures revealed 22 adults (30.5%)– 18 women and 3 men—positive to CD: later, they were hospitalized for disease staging and treatment. All the participants affected by CD came from the Departments of Santa Cruz and Cochabamba, the Bolivian areas in which the disease is more widespread [19, 20, 41]. Communicating screening results generated a profound feeling of shame in the affected participants.

As said, the stigma associated with the disease clearly appeared during diagnosis communication and during or after hospitalization, thus making the disease as permeated by a deep stigma. The following narratives are very illustrative:

Having Chagas, means being born in the country, in poor houses, because only in poor houses *vinchuca* can live

(Alfredo—March 3, 2018).

I come from a very poor background, I lived in the countryside, I was used to sleep on the ground, on banana leaves and I had no “light” at home. When we lit up the torch at night we used to see all the *vinchucas* walking on the walls, they were full of blood and surely they had stung the whole family. I cannot believe I lived like that, but now I am in Italy and I don’t want to think about it anymore

(Roland—March 8, 2018).

I do house cleaning, but how do I get to the hospital? My employers are very particular, as soon as they see that I have a cold they will not let me back in the house because they say that I am infected

(Carlita—April 29, 2018).

### Barriers to health: The weight of the Italian language, its culture and its health services

Despite many years spent in Italy (Table 3), the use of the Italian language by the interviewees was limited to the basic “technical” communication related to daily work activities.

**Table 3:** Distribution of patients included in anthropological study on Chagas Disease 2016–2018 on the basis of their permanence in Italy.

Year of arrival in Italy	N°	%
0 to 2 years ago (2018–2017)	4	5.6
3 to 5 years ago (2016–2014)	2	2.8
6 to 9 years ago (2013–2010)	8	11.1
10 to 20 years ago	48	66.7
More than 20 years ago (Before 1999)	4	5.5
No answer	6	8.3

Interpersonal relationships are limited to other members of the local Bolivian community, family members residing in Bolivia and in other European countries. Out of these contexts, interaction is minimal, even when necessary to exercise one’s rights, including health rights. Indeed, most of the interviewed people had a health card expired for years and did not know how to access the National Health Service; in the event of acute situations they just referred to the territorial emergency rooms.

In this scenario, it is understandable the skepticism that people showed when they were offered a free health screening to look for a disease and more, in the absence of symptoms. While the medical doctor insisted for prevention, this precise concept was causing suspicion in the recruited participants: a health intervention causing inability to work for one or more days was useless, especially in the absence of pain. Moreover, screening given freely accentuated their suspiciousness thus raising doubts of being “used” for scientific purposes instead of representing a reassuring element. Overall, this diffidence has accompanied all the research phases, including the moment of signing the informed consent, which often required extensive explanation by both the medical doctor and the anthropologist.

## Discussion

Our findings show that strengthening the fight against Chagas disease in Italy is no longer just a biomedical act. To intervene on the disease at a diagnostic and treatment level, we observed how decisive it was to consider aspects such as: fragile socio-economic conditions, difficult access to the Italian National Health Service due to linguistic, economic and legal barriers and precarious working conditions that makes it difficult to undergo a medical examination, and even more problematic to leave the workplace for hospitalization in case of administration of Chagas Disease treatment. Moreover, we also observed how the “explanatory models" of *illness* (perception of suffering according to the patient)—which revealed the stigma of poverty as a deterrent to treatment—and of *disease* (interpretation of suffering according to biomedicine) [30] have significantly interfered with the possibility of healing and curing [42].

### The territorial dislocation of the stigma

The dichotomous nature of Chagas and its various implications—ecological, social, political, economic and legislative—are the reasons why CD appears full of ambiguity and complexity. Furthermore, its predominantly asymptomatic character raises its invisibility whereas it does not weaken the deep stigma it is permeated by. To this regard, during the study, it appeared clear that all the participants tried to forget the disease and leave it in the shadows to hide the poor contexts of origin where they could have potentially become infected. The health care project of which the ethnographic study is part recalled the risk of the disease among people who live in Italy and, for these reasons, it inspired many contrasting feelings and attitudes that found their evidence in both recruitment and compliance difficulties that were observed and described (for example, the project was normally positively welcomed but then laboratory tests and subsequent appointments were often escaped).

In this context, the anthropological research pointed out the implicit unsaid about Chagas Disease and the stigma’s semantic codes that were causing poor compliance. This latter aspect clearly emerged during the research and had a strong impact on the completeness of the collected data. Haste and anxiety were common feelings during the procedure to access our outpatient clinic. Some participants, once arrived in the waiting room, leaved the Centre because it was impossible to wait 30 minutes to complete registration before meeting us. The fear of losing their job and the specter of not being able anymore to send economic remittances to their families became an important health barrier. For all of them, the anthropologist had to contact and plan easy and individualized accesses during non- working hours, including Saturday and Sundays. This entailed a complex negotiation, positively resolved, of socio-cultural and biomedical codes between participants and medical doctor/anthropologist.

Additionally, the above-described fear of losing their job also had a major impact on that related to receiving the diagnosis. We faced several difficulties in planning the appointment to communicate the results of laboratory tests, since a positive result could mean having to deal with other encounters to be scheduled for supplementary diagnostic tests, therapy and follow-ups.

### Cultural metaphors of poverty

Having Chagas means being born in the countryside, in poor houses, because *vinchuca* can live only in poor houses. Participants here hide their past living conditions, not only to the other members of the Bolivian community but, frequently, also to their own relatives. In this sense, it was extremely important for those diagnosed with CD (and their families) to secrete hospitalization from their compatriots, though sometimes this could have been difficult to achieve especially for those who lived with other Bolivians in the same housing occupation. In this regard, it is useful to know that the occupied buildings that hosted many members of the Bolivian community do not always have single apartments for each family, but functional spaces shared by several families, with one bathroom available for numerous people and a high level of proximity and promiscuity. The impossibility to hide one’s absence or a family member’s for the entire duration of the hospitalization, represented a profound resistance to treatment.

At the same time, we started to observe frequent alliances and conflicts, rising within and between communities both during interviews and social events: in most cases, participants used to mutually blame for having built up a false and fictitious social status, claiming—for example—to have grown up in a “city context” while real origins were to be found in the countryside and its poor environment. Having CD actually reminded participants of their precarious conditions as well as the poor context they came and from which they had escaped in the hope of rebuilding a new identity in a completely new reality, even if under the unsatisfactory condition of underemployed migrants.

According to this, we can undoubtedly say that Chagas disease threatened the construction of a self that was socially functional before, but which seems now to hold hostage the possibility of cure.

The need for hospitalization of positive participants had also a more practical, not less important consequence, related to the risk of losing the job. On one hand, the impossibility to stay away from work for a few days (most women are engaged in the care of the elderly and children); on the other, the fear of stigma by the employers too (if they do not know Chagas disease they may think it is a contagious pathology able to be transmitted to their family members as well).

### Issues of multidisciplinary approach

Our findings showed us that in our contemporary societies, crossed by such massive migration flows, the construction of multidisciplinary care spaces in public health, able to integrate the knowledge of social sciences—in particular medical anthropology—with that of biomedicine, is a health strategy that reveals benefits both for migrants and the host society. Indeed, the lack of familiarity with the categories of “illness” and “disease”, which are characteristic of a particular system of thought, and other etiological and therapeutic registers, are at the origin of many of the difficulties reported by health care professionals.

In our study, the effects of anthropological research were intended to aimed at directly intervening in the clinical project. Based on the results obtained, in fact, hours and days of medical examinations were modified, thus causing a structural intervention in a public health structure. Therefore, communication between doctor and patient was modified if hospitalization was provided, favoring the emergence of individual and familiar resistance to treatment due to the social consequences (especially when cohabitation made it explicit) of being affected by Chagas, therefore poor people from Bolivian rural areas. Additionally, all interviews, conversations and communications were in Spanish: the use of the native language was another key piece of the overall framework, since it gave staff members more chances to investigate and gather effective and additional evidence of how participants do experience Chagas illness [43].

During this research-action, clinicians and anthropologists succeeded in holding together both the clinical and ethnographic methods, thus applying what the ethnopsychiatrist Georges Devereux used to call “the double discourse". By this label, it is meant clinician’s and participant’s discourse [44], which are now considered as complementary items in all existing care practices.

### Limitations

Our study has several limitations. Firstly, only 72 participants were enrolled. From the beginning of the research, we found serious difficulties in recruiting participants due to several factors: precarious working conditions prevented them from taking appointments and keeping them; important stigma affecting Chagas disease in the communities of origin and in Bolivian communities in Rome. In this case, it was complex to maintain a continuous relationship with people who did not want to be hospitalized when necessary and for a period of time avoided any contact, even by telephone, skipping the appointments both with the anthropologist and the doctor.

Secondly, the anthropologist recruited the patients in Bolivian roman community for the clinical project on neglected diseases before and, after that, for the ethnographic research. This procedure, on the one hand facilitated project activities, because participants were at the outpatient clinic to be to be taken care of, but, on the other hand, the study was affected by patients’ difficulties with Chagas’ disease and the treatment paths related to it.

Finally, the anthropologist translated from Italian to Spanish and *vice versa* also during medical examinations and laboratory tests. This aspect certainly facilitated interview administration. However, this also probably created the conditions under which the participant-patient hardly would have escaped such a request.

## Supporting information

S1 Interview Semi-structured and unstructured interview in English. (DOC)

S2 Interview Semi-structured and unstructured interview in Italian. (DOC)
