# Supplementary material for: Anthropological study on Chagas Disease: Sociocultural construction of illness and embodiment of health barriers in Bolivian migrants in Rome, Italy
Source: PLoS One. 2020 Oct 16;15(10):e0240831. doi: 10.1371/journal.pone.0240831 (PMC7567347; doi:10.1371/journal.pone.0240831)
Supplement: S1 Interview — (DOC) [file pone.0240831.s001.doc]

1. Before your participation in the project, have you ever heard about Chagas Disease? **Yes No**
2. If yes, where did you hear about it?
3. Is it a contagious disease? **Yes No**
4. If yes, how do you think it can transmit?
5. Have you ever had any health problems related to Chagas Disease? **Yes No**
6. Has any member of your core family or extended ones had health problems? **Yes No**
7. If so, what are they:
8. Did you or your family members ever visit the doctor because of Chagas Disease? **Yes No**
9. If not, why? (*Analyze costs and social stigma*)
10. If yes, what did the doctor tell you about it?
11. What kind of treatment did the doctor prescribe you/your family members?
12. Did you follow the treatment continuously or did you stop it?
13. If you stopped it, why?
14. Who or what can cause Chagas Disease (*Analyze magic – religious aspects, as well as socio – cultural and socio – economic issues*)
15. What do you think might happen if the disease affects you?
16. What do people you know think about it? (*Analyze* *social and family related imaginary; prejudice, eventual isolation, contagion*)

**IN CASE OF POSITIVE PATIENTS:**

1. Since the Disease has affected you, how has your life changed?
2. Do you work the same as before? **Yes No**
3. Is your relationship with family and friends still unvaried?     **Yes No**
4. Is hospitalization a problem for your family? **Yes No** (If yes, why)
5. Is hospitalization a problem for your job? **Yes No** (If yes, why)
