# Supplementary material for: Anthropological study on Chagas Disease: Sociocultural construction of illness and embodiment of health barriers in Bolivian migrants in Rome, Italy
Source: PLoS One. 2020 Oct 16;15(10):e0240831. doi: 10.1371/journal.pone.0240831 (PMC7567347; doi:10.1371/journal.pone.0240831)
Supplement: S2 Interview — (DOC) [file pone.0240831.s002.doc]

1. Aveva mai sentito nominare prima del progetto la malattia di Chagas? Si No
2. (se sì) Dove?
3. È una malattia contagiosa? Si No
4. Se sì, come pensa si trasmetta?
5. Ha avuto mai problemi di salute per a causa del Chagas? Si No
6. Qualche membro della sua famiglia nucleare o estesa ha mai avuto problemi di salute? Si No
7. Se sì, quali.
8. È stata/membri della sua famiglia sono stati/ dal medico per il Chagas? Si No
9. Se no, perché? (Indagare costi e stigma sociale)
10. Se sì, Cosa le/vi ha detto il dottore?
11. Cosa le/vi ha prescritto?
12. Ha effettuato tutto il trattamento o lo ha interrotto?
13. Se lo ha interrotto, perché?
14. Da cosa o chi può essere causato il Chagas? (Indagare aspetti magico-religiosi, socio-culturali, socio-economici)
15. Cosa pensa possa accadere con questa malattia?
16. Cosa ne pensa la gente che conosce? (Indagare aspetti dell’immaginario sociale e familiare; pregiudizio, eventuale isolamento, contagio)

**Se persona positiva**

1. Da quando ha questa malattia, com’è cambiata la sua vita?
2. Lavora come prima? Si No
3. Le sue relazioni con amici e famiglia sono invariate? Si No
4. Il ricovero le crea problemi con la sua famiglia? Si No (Se sì perché)
5. Il ricovero le crea problemi con il lavoro? Si No (Se sì perché)
